# Mining Complex Ecological Patterns in Protected Areas: An FP-Growth Approach to Conservation Rule Discovery

**DOI:** 10.3390/e27070725

**Published:** 2025-07-04

**Authors:** Ioan Daniel Hunyadi, Cristina Cismaș

**Affiliations:** Department of Mathematics and Informatics, Faculty of Science, Lucian Blaga University of Sibiu, 550024 Sibiu, Romania; cristina.cismas@ulbsibiu.ro

**Keywords:** ecological information systems, fish species distribution modeling, association rule mining, entropy-based data analysis, complex ecosystem patterns, protected area management, knowledge discovery in databases

## Abstract

This study introduces a data-driven framework for enhancing the sustainable management of fish species in Romania’s Natura 2000 protected areas through ecosystem modeling and association rule mining (ARM). Drawing on seven years of ecological monitoring data for 13 fish species of ecological and socio-economic importance, we apply the FP-Growth algorithm to extract high-confidence co-occurrence patterns among 19 codified conservation measures. By encoding expert habitat assessments into binary transactions, the analysis revealed 44 robust association rules, highlighting interdependent management actions that collectively improve species resilience and habitat conditions. These results provide actionable insights for integrated, evidence-based conservation planning. The approach demonstrates the interpretability, scalability, and practical relevance of ARM in biodiversity management, offering a replicable method for supporting adaptive ecological decision making across complex protected area networks.

## 1. Introduction

The objective of this study is to develop a data-driven methodology that supports the sustainable conservation of fish species in Romania’s Natura 2000 protected areas. The purpose is to identify and analyze complex ecological relationships among conservation management actions through ecosystem modeling and the application of association rule mining (ARM), specifically the FP-Growth algorithm. By uncovering frequent co-occurrence patterns among 19 codified habitat measures, this research aims to provide actionable insights that can guide integrated, evidence-based, and adaptive ecological decision making.

Romania’s Natura 2000 network is a cornerstone of the European Union’s biodiversity strategy, encompassing approximately 20% of the national territory. These areas include a variety of freshwater habitats that are vital for the conservation of both endemic and migratory fish species [1]. In addition to their ecological significance, these sites hold socio-economic value, as they often overlap with traditional fishing communities and anthropogenically influenced landscapes [2].

This study targets 13 fish species—such as *Barbus barbus*, *Salmo trutta fario*, and *Rhodeus amarus*—chosen for their ecological sensitivity, conservation status under the EU Habitats Directive, and the availability of comprehensive long-term monitoring data [3,4]. Achieving and maintaining favorable conservation status for these species requires a holistic approach that involves evaluating critical habitat indicators, including water quality, substrate composition, vegetation cover, and anthropogenic pressures.

Using expert ecological assessments compiled over seven years, these evaluations are transformed into a binary decision matrix suitable for data mining. The FP-Growth algorithm is then applied to this matrix to uncover frequent, high-confidence associations among management measures. Traditional ARM metrics such as support, confidence, and lift are complemented by entropy-based measures—information gain and conditional entropy—to assess the informational strength and predictability of each rule.

This hybrid analytical approach aims to reveal both statistically significant and ecologically meaningful relationships between management actions. By doing so, it enhances the interpretability of conservation dependencies and supports strategic planning for ecosystem resilience. The findings serve as a replicable model for applying computational methods to biodiversity governance and contribute to the growing field of computational ecology.

The organization of this paper is structured to guide the reader through the development and application of a data-driven framework for conservation planning. The Related Literature Section reviews existing research on ecological modeling, the Natura 2000 network, and the application of data mining techniques in biodiversity conservation. The Association Rule Mining with Entropy-Based Perspectives Section introduces the FP-Growth algorithm and explains the integration of traditional and entropy-based metrics to enhance rule interpretation. The Methodology Section describes the data collection process, the transformation of expert ecological assessments into binary formats, and the implementation of the mining framework via RapidMiner. The Results Section presents the most significant association rules, interprets their ecological meaning, and visualizes their interdependencies through a thematic network graph. Finally, the Conclusions Section summarizes the key findings, highlights the contributions to computational ecology, and suggests directions for future research. The Appendix A provides a thematic classification of the rules to support practical conservation decision making.

## 2. The Related Literature

The integration of data-driven methodologies into ecological conservation has gained significant traction in recent years. The European Union’s Natura 2000 network, established under the Birds Directive and the Habitats Directive, underscores the importance of preserving biodiversity through designated protected areas. Achieving favorable conservation status for species within these areas necessitates adaptive management strategies informed by robust data analysis.

Association rule mining (ARM), particularly the FP-Growth algorithm, has emerged as a potent tool for uncovering hidden patterns within ecological datasets. Unlike traditional methods, FP-Growth efficiently identifies frequent itemsets without candidate generation, making it suitable for large and complex ecological data. Recent studies have applied ARM to various ecological contexts, demonstrating its versatility and effectiveness.

For example, a study by Jang et al. [5] introduced a region-based FP-Growth algorithm to analyze spatial data in the Internet of Things (IoT) environment, highlighting the algorithm’s adaptability to spatially explicit ecological data. Similarly, Bao et al. [6] proposed an improved evaluation methodology for mining association rules, enhancing the speed and accuracy of ARM in ecological applications.

In the realm of fish habitat modeling, recent advancements have focused on integrating various data sources to assess habitat suitability comprehensively. Park et al. [7] evaluated fish habitat suitability by simulating hydrodynamic and water quality factors via linked SWAT and HEC-RAS models, providing insights into habitat conditions under various environmental scenarios. Additionally, a study published in 2025 introduced the Fish Habitat Comprehensive Suitability (FHCS) indicator, which plays a pivotal role in evaluating fish spawning and egg hatching behavior.

These studies collectively underscore the growing importance of integrating advanced data analysis techniques, such as ARM and machine learning, into ecological research and conservation planning. By leveraging these tools, researchers and policymakers can develop more effective strategies for managing and preserving biodiversity within protected areas.

To ensure methodological rigor, the selection of fish species also considered their ecological representativeness, trophic roles, and sensitivity to environmental changes. This allowed for the development of a comprehensive model that captures inter-species and habitat-management interactions. Furthermore, the chosen species are indicative of the broader ecological health of their respective ecosystems, making them ideal proxies for evaluating the effectiveness of conservation and restoration measures [8,9].

The integration of high-quality ecological data with data mining techniques facilitates the extraction of meaningful patterns that can inform adaptive management. This approach aligns with contemporary conservation paradigms emphasizing evidence-based decision making, cross-disciplinary integration, and long-term ecosystem resilience [10,11].

To address the complexity of such ecological systems, this study introduces a cybernetic, data-driven modeling approach based on association rule mining (ARM) via the FP-Growth algorithm. ARM has proven effective for uncovering hidden patterns in large datasets across various domains, including ecology, because of its ability to detect statistically significant co-occurrences [12,13].

In our study, we analyzed conservation measures associated with 13 fish species from Natura 2000 sites in Romania. By transforming expert assessments into a structured, binary format, we apply FP-Growth to discover frequent co-occurrence patterns among management actions. This approach supports the development of integrated strategies aimed at enhancing species resilience and promoting ecosystem sustainability.

The analysis was implemented via RapidMiner, a powerful data mining platform known for its extensibility and user-friendly workflow design [14]. The use of such advanced analytical tools allows conservation practitioners and decision-makers to gain actionable insights, aligning conservation planning with evidence-based, adaptive management principles.

This research demonstrates how ARM can be leveraged to inform sustainable fish species management within protected areas. It contributes to the growing field of computational ecology, where data science and ecological knowledge intersect to address complex conservation challenges [10,15].

## 3. Association Rule Mining with Entropy-Based Perspectives

To uncover latent patterns and decision-relevant dependencies among conservation measures, we employed association rule mining (ARM) via the FP-Growth algorithm. ARM is a powerful data mining technique capable of extracting interpretable if–then rules from large transactional datasets. In the context of protected ecosystem management, such rules help identify frequent co-occurrences and conditional relationships between applied habitat measures, reflecting underlying ecological or administrative dependencies.

Unlike the Apriori algorithm, which performs iterative candidate generation, FP-Growth leverages a compact data structure known as the frequent pattern tree (FP-Tree) to efficiently identify itemsets without costly enumeration. This computational efficiency is particularly valuable when analyzing ecological datasets with high dimensionality and overlapping management actions.

To encode the expert-evaluated habitat data, we transformed the conservation measure assessments into binary transactions across seven years and 13 target fish species. Each transaction consisted of a set of habitat management measures rated as “good,” enabling the detection of positively associated actions. A minimum support threshold of 61% and confidence threshold of 95% were selected on the basis of empirical testing and expert validation, ensuring that the discovered rules were both frequent and highly reliable.

In addition to traditional ARM metrics—support, confidence, and lift—we integrate entropy-based measures to assess the informational complexity and predictive strength of the discovered rules:

- Information Gain (IG): Measures the reduction in uncertainty about the consequent given the antecedent. A high IG indicates a strong predictive rule with significant explanatory power.
- Conditional Entropy H (Y|X): Quantifies the residual uncertainty about a conservation measure (Y) when another measure (X) is known. Lower conditional entropy values signify more deterministic relationships and guide the prioritization of rules in management planning.
- Rule Entropy Profile: By aggregating rule-level entropy statistics, we evaluated the overall complexity landscape of the management network. This provides insight into which measures contribute to high-entropy (uncertain or flexible) versus low-entropy (stable or essential) decision pathways.

These entropy-based evaluations enhance the interpretability of the rules beyond frequency-based metrics. For example, while several rules may have high support, their conditional entropy values help differentiate between rules that truly reduce decision uncertainty and those that reflect broad but ambiguous associations.

Through this integration of classical ARM and entropy theory, we advance a dual analytical lens—capturing not only what co-occurs but also how much information is gained through those co-occurrences. This enriches the ecological interpretability and strategic utility of rule mining in complex conservation environments.

### 3.1. Overview of Association Rules

An association rule is generally expressed in the form X → Y, where X and Y are distinct itemsets with no shared elements (X ∩ Y = ∅). This implies that the occurrence of itemset X increases the probability of itemset Y also occurring. To assess the significance and reliability of each rule, several key metrics are used, including the following:

- Support:

$$Support\left(X\to Y\right)=\frac{Count(X\cup Y)}{Total\;Transactions}$$

This denotes the frequency with which X and Y occur together in the dataset.

- Confidence:

$$Confidence\left(X\to Y\right)=\frac{Count(X\cup Y)}{Count(X)}$$

It measures how often items in Y appear in transactions that contain X.

- Lift:

$$Lift\left(X\to Y\right)=\frac{Confidence(X\to Y)}{Support(Y)}$$

Lift assesses the independence of X and Y. A lift greater than 1 suggests a positive correlation.

These metrics help in filtering out statistically significant and ecologically relevant rules [12].

### 3.2. Application in Ecosystem Modeling

In this study, each sampling event, such as a fish catch or ecological survey conducted at a specific time and location, is treated as a transaction, with the observed fish species representing the items within that transaction. This approach allows for the discovery of co-occurrence rules that shed light on ecological relationships, such as species associations, habitat affinities, or trophic interactions. For example, a rule such as “Species A → Species B” may suggest the following:

- Shared environmental or habitat preferences.
- Possible mutualistic relationships.
- Behavioral tendencies such as schooling or synchronized migration.

These insights are particularly valuable for ecosystem-based management (EBM), providing evidence to support decisions related to habitat zoning, conservation prioritization, and the regulation of fishing activities [16,17].

### 3.3. Advantages of ARM in Conservation

Association rule mining (ARM) provides several distinct advantages over traditional ecological modeling approaches:

- Scalability: This method efficiently handles large-scale, high-dimensional ecological datasets.
- Interpretability: The resulting rules are straightforward and easily understood by ecologists and decision-makers.
- Flexibility: ARM does not rely on assumptions about underlying data distributions or predefined interactions.
- Exploratory Power: This is particularly well-suited for hypothesis generation and exploratory analysis [18].

These strengths make ARM a powerful tool for analyzing complex and dynamic ecological systems, including marine protected areas (MPAs) and biodiversity hotspots.

### 3.4. Advantages of the FP-Growth Algorithm

The FP-Growth algorithm is a highly efficient technique for identifying frequent itemsets, eliminating the need for candidate generation—a key limitation of earlier methods such as Apriori. Introduced by Han et al. [19], FP-Growth is designed for scalability and optimized performance, making it especially suitable for analyzing large and complex ecological datasets that involve numerous species and sampling events.

In this study, the FP-Growth algorithm was used to identify frequently co-occurring fish species across multiple sampling events within Romania’s protected aquatic ecosystems. This approach facilitates the detection of persistent species associations under specific environmental conditions, highlights potential indicator species valuable for ecological monitoring, and reveals rare yet ecologically significant co-occurrences that may point to previously unrecognized interactions.

Such insights support data-driven conservation actions, including species prioritization, zoning decisions, and assessments of human-induced pressures.

It is important to acknowledge the limitations of the FP-Growth algorithm. Building the FP-Tree can be memory-intensive, particularly when working with dense datasets. Additionally, the recursive nature of tree mining may lead to high computational demands in cases involving large numbers of distinct items. Moreover, FP-Growth is not inherently suitable for real-time or streaming data applications, although modified versions have been developed to address this constraint.

## 4. Methodology

This study’s methodological framework combines ecological data collection with advanced data mining methods to uncover patterns and relationships among management measures associated with fish species in protected areas. The approach involves three key stages—data acquisition, data preprocessing, and the application of the FP-Growth algorithm for association rule mining—all of which were conducted within the RapidMiner v. Studio 7 analytical environment.

### 4.1. Data Collection and Preparation

Ecological data for this study were gathered over a seven-year period through extensive fieldwork, expert input, and the analysis of historical records. The dataset encompasses 13 fish species of notable conservation and economic value, all located within selected Natura 2000 sites in Romania. For each species, a detailed ecological profile was developed, outlining key habitat features, biological requirements, and human-induced pressures. These profiles incorporated measurable indicators such as substrate composition, water flow velocity, dissolved oxygen levels, and the presence of structural elements such as riparian vegetation and oxbow lakes.

Experts assessed whether the current condition of each indicator met the criteria for favorable conservation status. This evaluation enabled the creation of binary decision matrices that reflected the necessity of specific management measures to maintain or enhance habitat quality. Indicators such as water quality, vegetation coverage, sedimentation, and anthropogenic disturbance were examined to determine the relevance of each measure.

These assessments were then encoded into binary variables for data mining: a value of 1 indicated that a particular management measure was recommended for a given species, whereas 0 indicated otherwise. The resulting transactional dataset consisted of rows representing individual species and columns representing the presence or absence of recommended management actions.

The methodological emphasis lies in leveraging this binary structure to reveal latent ecological patterns via association rule mining. The decision to focus on binary presence–absence data allowed for clear rule generation while maintaining ecological interpretability. The approach supports the broader objective of this study: to develop a replicable, data-driven decision-support framework capable of guiding conservation planning across species and protected habitats.

### 4.2. Management Measures

Nineteen distinct management measures were defined and codified (A–T) to facilitate computational processing. These measures encompassed a range of hydrological interventions—such as maintaining natural flow regimes and installing fish passages—as well as biological and regulatory strategies, including anti-poaching efforts and the reinforcement of vegetation buffers. Each fish species was linked to a specific combination of these measures, resulting in a structured transactional database suitable for applying association rule mining techniques.

Table 1 presents the codification of the 19 management measures used throughout the analysis. Each code corresponds to a specific intervention strategy aimed at improving or maintaining the favorable conservation status of target fish species within Natura 2000 sites.

Table 2 presents the relationships between the 13 fish species analyzed and the 19 codified management measures (A–T). Each cell in the matrix indicates whether a particular management action is deemed applicable (denoted by 1) or not applicable (denoted by 0) to a given species on the basis of ecological assessments and expert judgment. This matrix serves as the foundational dataset for the association rule mining carried out in this study.

### 4.3. Data Mining Framework

The data mining framework applied in this study was structured to systematically uncover frequent patterns and meaningful associations among management measures essential for the conservation of fish species in protected areas. By combining expert-curated ecological datasets with computational rule-mining methods, the framework facilitates the extraction of actionable insights that can inform strategic biodiversity management decisions.

This approach is built upon four interrelated components: (1) data acquisition and preparation; (2) transaction encoding and transformation; (3) frequent pattern mining via the FP-Growth algorithm; and (4) rule filtering, interpretation, and application. The layered architecture of the framework ensures not only transparency and reproducibility but also flexibility for adaptation to other ecological contexts or environmental datasets.

RapidMiner Studio was employed as the primary platform for implementing the data mining process and was selected for its robust workflow design capabilities, transparency, and extensibility [14]. Within RapidMiner, the dataset underwent preprocessing steps, including attribute type conversion, removal of redundancies, and transformation into a transactional format compatible with the FP-Growth algorithm.

The operator-based design of RapidMiner enables a modular and well-documented process, with the “FP-Growth” operator functioning as the core analytical component embedded in a chain of operators responsible for data import, validation, and visualization.

FP-Growth was selected for its proven efficiency in identifying frequent itemsets without the need for iterative database scanning. Instead, it constructs a compact FP-Tree (frequent pattern tree) to aggregate shared transaction patterns, significantly accelerating the mining process even in complex and dense datasets [13].

**Figure 1:**
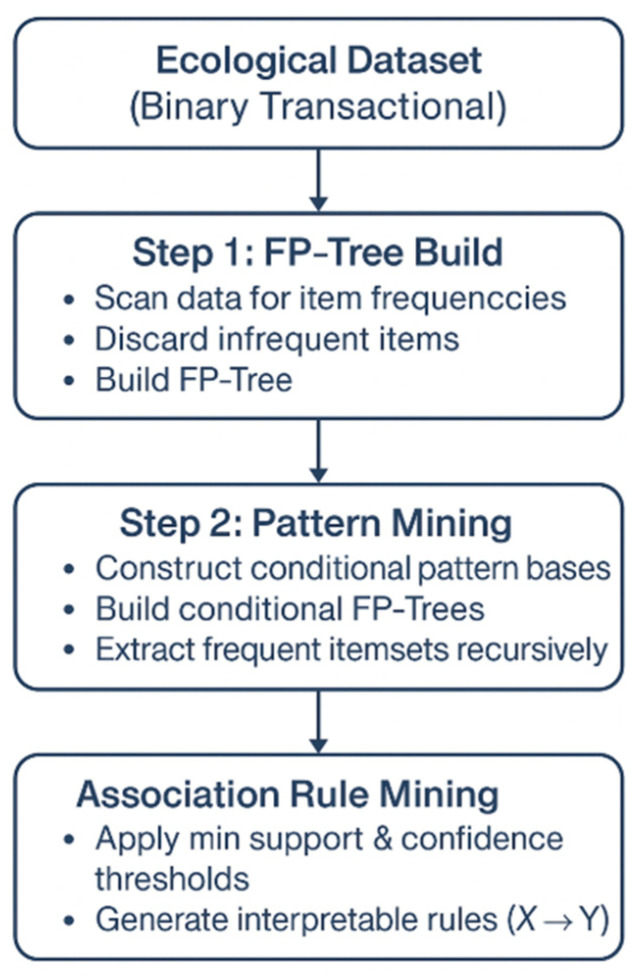
Simplified schematic of the FP-Growth algorithm used in this study.

As illustrated in Figure 1, the framework includes binary encoding of expert assessments, construction of the FP-Tree, extraction of frequent itemsets, and generation of association rules. This end-to-end process enables the rapid identification of interdependent conservation actions, offering a scalable and data-driven solution for ecosystem management.

Key thresholds were as follows:

- Minimum Support: 61% (i.e., rules must apply to at least 8 of 13 species).
- Minimum Confidence: 95% (ensuring high rule reliability).

The 61% minimum support threshold—corresponding to at least 8 of the 13 fish species—was defined in consultation with domain experts to balance ecological representativeness and statistical relevance. This threshold ensures that the derived rules reflect patterns occurring across a majority of species, thus enhancing their generalizability and practical utility in conservation planning. It was not arbitrarily selected but rather informed by expert judgment and iterative testing during exploratory analysis.

The algorithm identified all frequent itemsets that met the support threshold and generated association rules of the form X → Y, where X and Y are disjoint sets of codified management measures.

From an initial space of over 1.5 million potential rules (calculated using R = 3d − 2d + 1 + 1, where d is the number of management measures), the algorithm extracted 573 rules meeting the support and confidence thresholds. Of these, 44 were considered highly actionable on the basis of the complexity and number of items in their premises and conclusions. These were filtered based on the following:

- The number of items in the premise and conclusion.
- Ecological interpretability.
- Practical implementability in conservation plans.

The selection of the most actionable rules from the total of 573 generated by the FP-Growth algorithm was guided by a multi-criteria filtering process, ensuring ecological relevance, statistical reliability, and practical implementability. The key criteria used are as follows:

- Number of items in premise and conclusion: Rules with multiple items in both the premise and the conclusion were prioritized. These multi-measure rules reflect more complex and realistic conservation scenarios, capturing synergistic relationships among habitat management actions.
- Ecological interpretability: Rules were assessed for their biological and ecological plausibility. Selected rules had to make ecological sense, and the co-occurring measures must reflect known habitat interdependencies or practical field observations from conservation biology.
- Practical implementability: Measures included in the rules were evaluated for their feasibility in real-world conservation contexts. Rules recommending highly specialized, rarely applied, or cost-prohibitive interventions were filtered out.
- Support and confidence thresholds: All retained rules had to meet or exceed *Minimum Support* of 61% (applicable to at least 8 out of 13 fish species) and *Minimum Confidence* of 95% (in most retained rules, 100% confidence was achieved). This ensured that rules were frequent and reliable, representing meaningful patterns across the majority of species studied.
- Redundancy elimination: Redundant or highly similar rules (e.g., permutations of the same measures) were removed to avoid repetition and ensure conceptual uniqueness in the final rule set.
- Entropy-based informational value: Although not explicitly stated in every rule filter, rules that provided high information gain or low conditional entropy were implicitly favored. These rules reduced uncertainty and improved predictive clarity for decision-makers.

A total of 44 rules were retained for further analysis. Each rule offers actionable insights indicating that, if certain management measures are required for a species, others are likely to be beneficial as well.

The final rules are intended to support conservation planning by providing evidence-based suggestions for complementary management actions. For example, if habitat restoration (measure A) is applied, the rules may recommend simultaneous enforcement against poaching (E) and vegetation buffer protection (F).

This integration of data mining with ecological modeling represents a scalable approach for adaptive management under uncertainty and offers a replicable model for biodiversity analytics in other protected areas.

The analysis was guided by two key thresholds: a minimum support of 61%, meaning that a rule must be applicable to at least 8 out of the 13 fish species, and a minimum confidence of 95%, ensuring high reliability of the resulting associations. The 61% support threshold was not arbitrarily chosen; rather, it was established in consultation with domain experts to strike an appropriate balance between ecological representativeness and statistical significance. This level was selected to ensure that the derived rules capture patterns that are relevant to the majority of species studied, thereby enhancing their generalizability and practical value for conservation planning. The threshold was validated through expert judgment and iterative testing during the exploratory phase of the analysis.

Using these parameters, the FP-Growth algorithm identified all frequent itemsets that satisfied the minimum support criterion and generated association rules of the form X → Y, where X and Y represent disjoint sets of codified management measures. From an initial search space of more than 1.5 million potential rules—calculated via the formula $R=3^{d}-2^{d+1}+1$, where d is the number of management measures—the algorithm distilled a total of 573 rules that met both the support and confidence thresholds.

To ensure both ecological relevance and practical utility, a filtering process was applied to retain only the most actionable rules. This process considers several criteria, including the number of items in the premise and conclusion, the ecological interpretability of the rule, and its feasibility for implementation in real-world conservation contexts. Ultimately, 44 rules were selected for further analysis.

Each of these rules offers insights into complementary management measures, suggesting, for example, that, if habitat restoration actions (e.g., measure A) are recommended for a species, additional actions such as poaching control (E) and vegetation buffer preservation (F) are also likely to be beneficial. These associations provide a data-driven foundation for designing integrated conservation strategies.

By combining data mining techniques with ecological modeling, this framework delivers a scalable, adaptive approach to conservation planning under uncertainty. Moreover, it presents a replicable model for biodiversity analytics that can be applied to other protected area networks.

## 5. Results

This section presents the results of applying the FP-Growth algorithm to uncover association rules among management measures related to thirteen fish species inhabiting Natura 2000 protected areas. The primary objective is to support sustainable conservation strategies by identifying interdependent actions that, when implemented together, contribute to the long-term preservation of these species.

The analysis was based on management data for thirteen fish species, incorporating nineteen distinct management measures labeled from A to T. These data were derived from expert evaluations and extensive long-term field observations. To ensure both statistical robustness and ecological validity, the FP-Growth algorithm was applied within the RapidMiner environment using a minimum support threshold of 61% (i.e., applicable to at least 8 of the 13 species) and a minimum confidence threshold of 95%.

From a theoretical space of 8191 potential itemset combinations derived from 13 species and 19 management measures, the FP-Growth algorithm efficiently reduced the rule set to 573 associations that met the defined thresholds. Among these, 44 rules were selected for their practical significance—each featuring premises and conclusions that include at least three management actions. These rules represent strong and consistent dependencies between clusters of conservation measures.

Assuming that the full set of management measures is denoted as MM = {A, B, C, D, E, F, G, H, I, J, K, L, M, N, O, P, R, S, T} and that the database S contains records for thirteen fish species, the analysis was conducted using the previously defined thresholds. The resulting association rules all exhibit a confidence level of 100% and meet the 61% minimum support criterion.

The rules retained for detailed interpretation are those with multi-measure premises and conclusions, as they provide the most actionable guidance. These findings offer valuable insights into the co-occurrence of effective management strategies and support the design of holistic, evidence-based conservation plans.

The results obtained are presented in Table 3.

Each row corresponds to a rule premise. All rules in the selected sample show high confidence (1.0), with varying support across species. This illustrates the strength and reliability of conservation action dependencies.

There are several combinations of management measures that significantly influence other management measures that can be applied to the same fish species.

Let us also demonstrate an association rule that holds 63% of the time, starting from a premise of four management measures that lead to three other management measures. For example, if the following management measures are applied to a species:

- G: Prohibition of the abandonment of any type of waste in the riverbed and in the wetland areas adjacent to watercourses.
- A: Preservation of the natural morphodynamics of riverbeds, recommending the prohibition of hydraulic constructions/works that modify the flow velocity regime and the composition of the riverbed substrate.
- F: Preservation of natural vegetation corridors (trees, shrubs, and herbaceous plants) with widths of at least 25–100 m on both banks to ensure the necessary plant residues for reproduction.
- D: During the breeding period, fishing and activities that disturb riverbed sediment should be prohibited.

Sixty-three percent of the time, the following management measures are valid:

- T: Implementing an integrated monitoring system for ichthyofauna by qualified/specialized personnel;
- E: In all river sections of interest, illegal fishing activities are very intense and almost permanent, requiring more effective control;
- C: Exploiting mobile aggregates from the riverbed, specifically removing the sand substrate, should not be allowed to preserve the habitat characteristics of this species.

**Figure 2:**
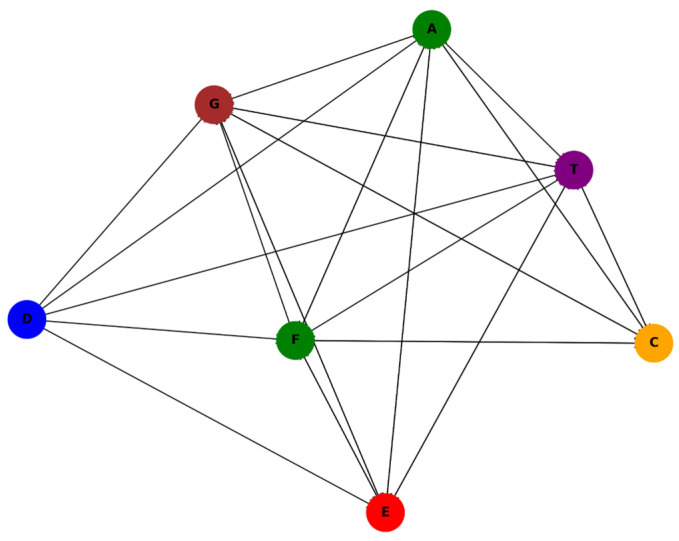
Network graph of association rules among management measures, color-coded by conservation theme.

In Figure 2, a thematic network graph is presented of all 44 association rules. Each node (management measure) is color-coded by its ecological theme:

- Green: Habitat restoration (e.g., A, F).
- Orange: Substrate protection (C).
- Blue: Breeding and reproduction-related genes (D).
- Red: Anti-poaching and enforcement (E).
- Brown: Pollution and environmental control (G).
- Purple: Monitoring and evaluation (T).

Each node represents a management action; directional edges (arrows) indicate rule-based dependencies (Premises → Conclusions). The node color reflects the ecological theme—habitat (green), enforcement (red), pollution control (brown), etc. This visualization highlights the interlinkages and synergistic patterns in multi-species conservation strategies.

The ease of interpretation and high speed of classification for new instances are just some of the key advantages of association rule-based classification.

The rules obtained can help decision-makers (managers, biologists, ecologists, government agencies, NGOs, etc.) in the decision-making process regarding the management measures to be applied for the conservation of certain fish species. For example, if measurements indicate that certain management measures need to be applied, the association rules obtained through the previously described model can suggest other management rules that can contribute to the conservation of the species.

To enhance the interpretability and practical relevance of the extracted association rules, the 44 high-confidence rules were thematically grouped according to the ecological focus of their associated management measures. These themes include habitat restoration, ant poisoning, pollution control, substrate protection, breeding regulation, and monitoring. Each rule often involves measures that span multiple themes, reflecting the interconnected nature of conservation challenges in protected freshwater ecosystems. By organizing the rules in this way, stakeholders can more readily identify clusters of synergistic actions that support specific conservation goals. For example, many rules highlight the consistent co-occurrence of habitat restoration efforts (e.g., preserving river morphodynamics and riparian vegetation) with anti-poaching controls and monitoring strategies, reinforcing the need for integrated, multi-faceted management approaches.

The association rules discovered among conservation management measures provide practical, data-informed guidance for enhancing the conservation of specific fish species in Romania’s Natura 2000 protected areas. These rules can be applied in real-world conservation planning as follows:

- Integrated Management Planning: Association rules such as A, F, T → G, E, and C. These rules imply that when habitat restoration (A), riparian buffer preservation (F), and monitoring (T) are required for a species, it is highly likely that pollution control (G), anti-poaching enforcement (E), and substrate protection (C) should also be implemented. When conservationists identify the need for habitat restoration in a species such as Salmo trutta fario, they can simultaneously plan for complementary measures such as stricter anti-poaching controls and sediment regulation, thereby ensuring a holistic response rather than piecemeal interventions.
- Decision Support for Uncertain or New Cases: In cases where field data are incomplete or where species-specific assessments are lacking, the rules can infer additional necessary actions on the basis of observed patterns in ecologically similar species. For a lesser-known species such as *Romanogobio albipinnatus*, if expert judgment recommends actions A and F, the model may suggest adding G, E, and C, even if direct evidence is not yet fully documented. This reduces decision-making uncertainty and fills data gaps via learned patterns.
- Conservation Policy Design and Prioritization: The rules help identify clusters of management actions that frequently co-occur across multiple species, enabling agencies to bundle policies for efficiency. If 8 out of the 13 fish species benefit from the same package of measures (e.g., A + F + E + T), environmental managers can prioritize these as core actions in multi-species conservation strategies, optimizing both cost and impact.
- Ecosystem-Based Zoning and Restoration: Some rules suggest how one type of intervention (e.g., anti-poaching) is tightly linked with others (e.g., vegetation restoration), supporting ecosystem-wide planning. In a degraded river stretch where poaching is rampant, rules might recommend not only law enforcement but also actions such as riparian vegetation restoration (F) and aggregate removal bans (C). This encourages multi-dimensional restoration beyond symptom-based responses.
- Monitoring and Evaluation (M&E) Frameworks: Rules that involve measuring T (Monitoring) show how it consistently co-occurs with other interventions, highlighting its role in adaptive management. Conservation programs can design M&E protocols to track the effectiveness of grouped measures specifically, allowing for the evidence-based iteration of conservation plans over time.

Appendix A presents these rules by theme group, enabling targeted insights into which combinations of measures are most frequently and effectively recommended together across the analyzed fish species.

## 6. Conclusions

This study presents a novel, data-driven approach to ecosystem management by revealing latent conservation dependencies among habitat measures for fish species in protected areas. Through the application of the FP-Growth algorithm to structured ecological monitoring data, we extracted interpretable association rules that reveal the underlying informational structure and management interdependencies within Natura 2000 sites. These rules not only quantify frequently co-occurring conservation actions but also expose complex, non-obvious linkages that contribute to the emergent behavior of aquatic ecosystems.

Our approach contributes to the field of computational ecology by demonstrating how rule mining methods, rooted in information theory, can transform discrete ecological observations into high-value knowledge for adaptive management. The discovered patterns can be interpreted as low-entropy, high-confidence decision pathways within a broader, complex conservation space. This reduces uncertainty in planning and enhances the system-level understanding of species–habitat interactions.

Furthermore, this work lays the foundation for integrating entropy-based metrics in future analyses—for instance, measuring rule diversity, ecosystem response variability, or management information gain. By extending association rule mining into an entropy-aware framework, protected area managers could better anticipate system responses under resource constraints or shifting ecological baselines.

This study illustrates the potential of frequent pattern analysis as a scalable and explainable tool for ecological complexity reduction, knowledge extraction, and multi-species conservation planning. Future work may incorporate temporal dynamics, entropy-based complexity metrics, and socio-ecological modeling to deepen this computational paradigm for biodiversity governance.

## Figures and Tables

**Table 1:** Codification of management measures.

Management Measures	Code
Maintain the natural morphodynamics of riverbeds by restricting constructions that alter flow regimes or substrate composition.	A
Install advanced fish ladder systems to address flow discontinuities and improve fish migration.	B
Ban the extraction of mobile aggregates to preserve the structural integrity of aquatic habitats.	C
Restrict fishing activities and sediment-disturbing operations during critical breeding periods.	D
Strengthen enforcement efforts to combat poaching in vulnerable river sections.	E
Protect and maintain natural vegetation corridors, 25–100 m wide, along riverbanks.	F
Prohibit the dumping of waste in riverbeds and adjacent wetland areas.	G
Preserve natural riparian vegetation to mitigate erosion and regulate sediment transport.	H
Prevent disruption of minor riverbed structures and regulate the spacing of sediment extraction sites.	I
Safeguard oxbow lakes and nearby wetlands as essential aquatic habitats.	J
Ensure adequate water quality and oxygen levels to support mollusk populations.	K
Preserve the natural morphodynamics of smaller river channels to facilitate mollusk reproduction.	L
Implement targeted measures to control pollution and maintain ecosystem health.	M
Support the presence and viability of *Unio* and *Anodonta* mollusk species within aquatic habitats.	N
Maintain a stable, permanent water regime in small rivers while minimizing human impact.	O
Conserve the natural hydrological patterns across all water bodies.	P
Integrate fish passage infrastructure into hydrotechnical projects to ensure species connectivity.	R
Manage invasive fish species through selective and targeted fishing practices.	S
Conduct regular ichthyofauna monitoring with trained and specialized personnel.	T

**Table 2:** Codified management measures and their distributions for the fish species analyzed.

Management Measures/Species	A	B	C	D	E	F	G	H	I	J	K	L	M	N	O	P	R	S	T
*Romanogobio kesslerii*	1	1	1	1	1	1	1	0	0	0	0	0	0	0	0	0	0	0	1
*Rhodeus amarus*	0	0	0	1	1	1	1	0	1	1	1	1	1	1	0	0	0	0	1
*Zingel zingel*	1	1	1	1	1	1	1	0	0	0	0	0	0	0	0	0	0	0	1
*Barbus meridionalis*	1	1	1	1	1	1	1	1	0	0	0	0	0	0	1	0	0	0	1
*Sabanejewia aurata*	1	0	0	0	0	0	1	0	0	0	0	0	0	0	0	0	0	0	1
*Eudontomyzon danfordi*	1	0	1	1	1	1	1	0	0	0	0	0	1	0	1	1	1	1	1
*Romanogobio albinnatus*	1	1	1	0	1	1	1	1	0	0	0	0	0	0	0	0	0	0	1
*Cottus gobio*	1	0	1	1	1	1	1	1	0	0	0	1	1	0	0	1	1	1	1
*Pelecus cultratus*	1	1	1	1	0	0	1	0	0	0	0	0	0	0	0	0	0	0	1
*Barbus barbus*	1	1	1	1	1	1	1	1	0	0	0	0	1	0	1	0	0	0	1
*Alburnus alburnus*	1	1	1	1	1	1	1	1	0	0	0	0	1	0	1	0	0	0	1
*Alburnoides bipunctatus*	1	1	1	1	1	1	1	1	0	0	0	0	1	0	1	0	0	0	1
*Salmo trutta fario*	1	0	1	1	1	1	1	1	0	0	0	1	1	0	0	1	1	1	1

**Table 3:** Obtained association rules after applying the FP-Growth algorithm.

Nr. Crt.	Premises	Conclusion	Support	Confidence
1	T = 1, A = 1, F = 1	G = 1, E = 1, C = 1	0.727272727	1
2	G = 1, A = 1, F = 1	T = 1, E = 1, C = 1	0.727272727	1
3	T = 1, A = 1, E = 1	G = 1, F = 1, C = 1	0.727272727	1
4	G = 1, A = 1, E = 1	T = 1, F = 1, C = 1	0.727272727	1
5	A = 1, F = 1, E = 1	T = 1, G = 1, C = 1	0.727272727	1
6	T = 1, F = 1, C = 1	G = 1, A = 1, E = 1	0.727272727	1
7	G = 1, F = 1, C = 1	T = 1, A = 1, E = 1	0.727272727	1
8	A = 1, F = 1, C = 1	T = 1, G = 1, E = 1	0.727272727	1
9	T = 1, E = 1, C = 1	G = 1, A = 1, F = 1	0.727272727	1
10	G = 1, E = 1, C = 1	T = 1, A = 1, F = 1	0.727272727	1
11	A = 1, E = 1, C = 1	T = 1, G = 1, F = 1	0.727272727	1
12	F = 1, E = 1, C = 1	T = 1, G = 1, A = 1	0.727272727	1
13	A = 1, F = 1, D = 1	T = 1, G = 1, E = 1	0.636363636	1
14	A = 1, E = 1, D = 1	T = 1, G = 1, F = 1	0.636363636	1
15	A = 1, F = 1, D = 1	T = 1, G = 1, C = 1	0.636363636	1
16	F = 1, D = 1, C = 1	T = 1, G = 1, A = 1	0.636363636	1
17	A = 1, E = 1, D = 1	T = 1, G = 1, C = 1	0.636363636	1
18	E = 1, D = 1, C = 1	T = 1, G = 1, A = 1	0.636363636	1
19	F = 1, D = 1, C = 1	T = 1, G = 1, E = 1	0.636363636	1
20	E = 1, D = 1, C = 1	T = 1, G = 1, F = 1	0.636363636	1
21	A = 1, F = 1, D = 1	T = 1, E = 1, C = 1	0.636363636	1
22	A = 1, E = 1, D = 1	T = 1, F = 1, C = 1	0.636363636	1
23	F = 1, D = 1, C = 1	T = 1, A = 1, E = 1	0.636363636	1
24	E = 1, D = 1, C = 1	T = 1, A = 1, F = 1	0.636363636	1
25	A = 1, F = 1, D = 1	G = 1, E = 1, C = 1	0.636363636	1
26	A = 1, E = 1, D = 1	G = 1, F = 1, C = 1	0.636363636	1
27	F = 1, D = 1, C = 1	G = 1, A = 1, E = 1	0.636363636	1
28	E = 1, D = 1, C = 1	G = 1, A = 1, F = 1	0.636363636	1
29	A = 1, F = 1, D = 1	T = 1, G = 1, E = 1, C = 1	0.636363636	1
30	T = 1, A = 1, F = 1, D = 1	G = 1, E = 1, C = 1	0.636363636	1
31	G = 1, A = 1, F = 1, D = 1	T = 1, E = 1, C = 1	0.636363636	1
32	A = 1, E = 1, D = 1	T = 1, G = 1, F = 1, C = 1	0.636363636	1
33	T = 1, A = 1, E = 1, D = 1	G = 1, F = 1, C = 1	0.636363636	1
34	G = 1, A = 1, E = 1, D = 1	T = 1, F = 1, C = 1	0.636363636	1
35	A = 1, F = 1, E = 1, D = 1	T = 1, G = 1, C = 1	0.636363636	1
36	F = 1, D = 1, C = 1	T = 1, G = 1, A = 1, E = 1	0.636363636	1
37	T = 1, F = 1, D = 1, C = 1	G = 1, A = 1, E = 1	0.636363636	1
38	G = 1, F = 1, D = 1, C = 1	T = 1, A = 1, E = 1	0.636363636	1
39	A = 1, F = 1, D = 1, C = 1	T = 1, G = 1, E = 1	0.636363636	1
40	E = 1, D = 1, C = 1	T = 1, G = 1, A = 1, F = 1	0.636363636	1
41	T = 1, E = 1, D = 1, C = 1	G = 1, A = 1, F = 1	0.636363636	1
42	G = 1, E = 1, D = 1, C = 1	T = 1, A = 1, F = 1	0.636363636	1
43	A = 1, E = 1, D = 1, C = 1	T = 1, G = 1, F = 1	0.636363636	1
44	F = 1, E = 1, D = 1, C = 1	T = 1, G = 1, A = 1	0.636363636	1

## Data Availability

The raw data supporting the conclusions of this article will be made available by the authors upon request.

## Abbreviations

The following abbreviations are used in this manuscript:

MDPI	Multidisciplinary Digital Publishing Institute
DOAJ	Directory of Open Access Journals
TLA	Three-letter acronym
LD	Linear dichroism

## Appendix A

**Thematic Group 1:** Anti-poaching, habitat restoration, monitoring, pollution control, and substrate protection.

Premise: T, A, F → Conclusion: G, E, C | Support: 0.727, Confidence: 1.00
Premise: G, A, F → Conclusion: T, E, C | Support: 0.727, Confidence: 1.00
Premise: T, A, E → Conclusion: G, F, C | Support: 0.727, Confidence: 1.00
Premise: G, A, E → Conclusion: T, F, C | Support: 0.727, Confidence: 1.00
Premise: A, F, E → Conclusion: T, G, C | Support: 0.727, Confidence: 1.00
Premise: T, F, C → Conclusion: G, A, E | Support: 0.727, Confidence: 1.00
Premise: G, F, C → Conclusion: T, A, E | Support: 0.727, Confidence: 1.00
Premise: A, F, C → Conclusion: T, G, E | Support: 0.727, Confidence: 1.00
Premise: T, E, C → Conclusion: G, A, F | Support: 0.727, Confidence: 1.00
Premise: G, E, C → Conclusion: T, A, F | Support: 0.727, Confidence: 1.00
Premise: A, E, C → Conclusion: T, G, F | Support: 0.727, Confidence: 1.00
Premise: F, E, C → Conclusion: T, G, A | Support: 0.727, Confidence: 1.00

**Thematic Group 2:** Anti-poaching, breeding regulation, habitat restoration, monitoring, and pollution control.

Premise: A, F, D → Conclusion: T, G, E | Support: 0.636, Confidence: 1.00
Premise: A, E, D → Conclusion: T, G, F | Support: 0.636, Confidence: 1.00

**Thematic Group 3**: Breeding regulation, habitat restoration, monitoring, pollution control, and substrate protection.

Premise: A, F, D → Conclusion: T, G, C | Support: 0.636, Confidence: 1.00
Premise: F, D, C → Conclusion: T, G, A | Support: 0.636, Confidence: 1.00

**Thematic Group 4**: Anti-poaching, breeding regulation, habitat restoration, monitoring, pollution control, and substrate protection.

Premise: A, E, D → Conclusion: T, G, C | Support: 0.636, Confidence: 1.00
Premise: E, D, C → Conclusion: T, G, A | Support: 0.636, Confidence: 1.00
Premise: F, D, C → Conclusion: T, G, E | Support: 0.636, Confidence: 1.00
Premise: E, D, C → Conclusion: T, G, F | Support: 0.636, Confidence: 1.00

**Thematic Group 5:** Anti-poaching, breeding regulation, habitat restoration, monitoring, and substrate protection.

Premise: A, F, D → Conclusion: T, E, C | Support: 0.636, Confidence: 1.00
Premise: A, E, D → Conclusion: T, F, C | Support: 0.636, Confidence: 1.00
Premise: F, D, C → Conclusion: T, A, E | Support: 0.636, Confidence: 1.00
Premise: E, D, C → Conclusion: T, A, F | Support: 0.636, Confidence: 1.00

**Thematic Group 6**: Anti-poaching, breeding regulation, habitat restoration, pollution control, and substrate protection.

Premise: A, F, D → Conclusion: G, E, C | Support: 0.636, Confidence: 1.00
Premise: A, E, D → Conclusion: G, F, C | Support: 0.636, Confidence: 1.00
Premise: F, D, C → Conclusion: G, A, E | Support: 0.636, Confidence: 1.00
Premise: E, D, C → Conclusion: G, A, F | Support: 0.636, Confidence: 1.00

**Thematic Group 7**: Anti-poaching, breeding regulation, habitat restoration, monitoring, pollution control, and substrate protection.

Premise: A, F, D → Conclusion: T, G, E, C | Support: 0.636, Confidence: 1.00
Premise: T, A, F, D → Conclusion: G, E, C | Support: 0.636, Confidence: 1.00
Premise: G, A, F, D → Conclusion: T, E, C | Support: 0.636, Confidence: 1.00
Premise: A, E, D → Conclusion: T, G, F, C | Support: 0.636, Confidence: 1.00
Premise: T, A, E, D → Conclusion: G, F, C | Support: 0.636, Confidence: 1.00
Premise: G, A, E, D → Conclusion: T, F, C | Support: 0.636, Confidence: 1.00
Premise: A, F, E, D → Conclusion: T, G, C | Support: 0.636, Confidence: 1.00
Premise: F, D, C → Conclusion: T, G, A, E | Support: 0.636, Confidence: 1.00
Premise: T, F, D, C → Conclusion: G, A, E | Support: 0.636, Confidence: 1.00
Premise: G, F, D, C → Conclusion: T, A, E | Support: 0.636, Confidence: 1.00
Premise: A, F, D, C → Conclusion: T, G, E | Support: 0.636, Confidence: 1.00
Premise: E, D, C → Conclusion: T, G, A, F | Support: 0.636, Confidence: 1.00
Premise: T, E, D, C → Conclusion: G, A, F | Support: 0.636, Confidence: 1.00
Premise: G, E, D, C → Conclusion: T, A, F | Support: 0.636, Confidence: 1.00
Premise: A, E, D, C → Conclusion: T, G, F | Support: 0.636, Confidence: 1.00
Premise: F, E, D, C → Conclusion: T, G, A | Support: 0.636, Confidence: 1.00
